# Enhanced therapeutic potential of antibody fragment via IEDDA-mediated site-specific albumin conjugation

**DOI:** 10.1186/s13036-024-00418-3

**Published:** 2024-04-04

**Authors:** Eun Byeol Go, Jae Hun Lee, Jeong Haeng Cho, Na Hyun Kwon, Jong-il Choi, Inchan Kwon

**Affiliations:** 1 School of Materials Science and Engineering, Gwangju Institute of Science and Technology (GIST), Gwangju, 61005 Republic of Korea; 2 ProAbTech, Gwangju, 61005 Republic of Korea; 3 Department of Biotechnology and Bioengineering, Interdisciplinary Program for Bioenergy and Biomaterials, Chonnam National University, Gwangju, 61186 Republic of Korea

**Keywords:** Single-chain variable fragment, Therapeutic antibody, Human serum albumin, Site-specific conjugation, Half-life extension

## Abstract

**Background:**

The use of single-chain variable fragments (scFvs) for treating human diseases, such as cancer and immune system disorders, has attracted significant attention. However, a critical drawback of scFv is its extremely short serum half-life, which limits its therapeutic potential. Thus, there is a critical need to prolong the serum half-life of the scFv for clinical applications. One promising serum half-life extender for therapeutic proteins is human serum albumin (HSA), which is the most abundant protein in human serum, known to have an exceptionally long serum half-life. However, conjugating a macromolecular half-life extender to a small protein, such as scFv, often results in a significant loss of its critical properties.

**Results:**

In this study, we conjugated the HSA to a permissive site of scFv to improve pharmacokinetic profiles. To ensure minimal damage to the antigen-binding capacity of scFv upon HSA conjugation, we employed a site-specific conjugation approach using a heterobifunctional crosslinker that facilitates thiol-maleimide reaction and inverse electron-demand Diels-Alder reaction (IEDDA). As a model protein, we selected 4D5scFv, derived from trastuzumab, a therapeutic antibody used in human epithermal growth factor 2 (HER2)-positive breast cancer treatment. We introduced a phenylalanine analog containing a very reactive tetrazine group (frTet) at conjugation site candidates predicted by computational methods. Using the linker TCO-PEG4-MAL, a single HSA molecule was site-specifically conjugated to the 4D5scFv (4D5scFv-HSA). The 4D5scFv-HSA conjugate exhibited HER2 binding affinity comparable to that of unmodified 4D5scFv. Furthermore, in pharmacokinetic profile in mice, the serum half-life of 4D5scFv-HSA was approximately 12 h, which is 85 times longer than that of 4D5scFv.

**Conclusions:**

The antigen binding results and pharmacokinetic profile of 4D5scFv-HSA demonstrate that the site-specifically albumin-conjugated scFv retained its binding affinity with a prolonged serum half-life. In conclusion, we developed an effective strategy to prepare site-specifically albumin-conjugated 4D5scFv, which can have versatile clinical applications with improved efficacy.

**Supplementary Information:**

The online version contains supplementary material available at 10.1186/s13036-024-00418-3.

## Introduction

Therapies using monoclonal antibodies (mAbs) have been successfully applied in the treatment of human diseases, such as tumors and inflammatory disorders [1]. Monoclonal antibodies exhibit exceptional specificity in binding to target molecules. The Fc region of mAb leads to the exceptionally long in vivo half-life by avoiding intracellular degradation through neonatal Fc receptor (FcRn)-mediated recycling [2] and triggers immune responses such as antibody-dependent cellular cytoxicicity. Nevertheless, cost-effective large-scale production of mAbs remains an unverified challenge when utilizing cultured mammalian cells or transgenic organisms. Additionally, mAbs often encounter limitations in penetrating solid tumors and may trigger undesired immune responses due to their Fc region [3–7]. To address these issues, alternative antibody formats have been explored, notably the single-chain variable fragment (scFv). The scFv can be efficiently generated using bacterial expression systems, providing a cost-effective production method compared to traditional mAb production [8, 9]. The scFv, encompassing the complete antigen-binding domains of full antibodies, retains specificity for an antigen without Fc-mediated immune responses. However, one critical drawback of the scFv is its very short half-life in vivo due to the lack of Fc region, which limits its therapeutic efficacy. Therefore, prolonging the serum half-life of scFv is important for the development of scFv-based therapeutics [10–13].

Various methods have been explored to prolong in vivo half-life, such as conjugation with polyethyleneglycol (PEG) and fusion to immunoglobulin binding protein/albumin binding protein [14–16]. However, PEG and non-human originated binding proteins exhibit potential immunogenicity issues and often reduce antigen binding affinity [17–25]. Therefore, developing alternative strategies to prolong the in vivo half-life of scFvs is still important. Human serum albumin (HSA) is notably characterized by an exceptionally extended half-life, exceeding three weeks, and known to be biocompatible and degradable in vivo [26]. In addition to these features, HSA is known to be a very promising serum half-life extender of therapeutic proteins [27–29]. Similar to mAbs, HSA exhibits exceptionally long serum half-life owing to FcRn-mediated recycling by endothelial cell [30–32].

In this study, to improve the pharmacokinetic profile of scFv, which has a short half-life, we performed bioconjugation of scFv with HSA. We chose HSA instead of mouse serum albumin (MSA) as a conjugation partner, because HSA has more relevance to clinical applications than MSA. However, it is noteworthy that the binding of HSA to mouse FcRn (mFcRn) is weaker than that of MSA [33]. Therefore, the limited serum half-life extension by HSA conjugation compared to MSA conjugation was expected in mice to determine the serum half-life. HSA can be fused to either N-terminus or C-terminus of scFv. Since either terminus is often involved in antigen binding, there is a potential risk of the greatly reduce antigen binding affinity. Furthermore, the conjugation of HSA to the site of scFv away from both termini, those termini are available for fusion to other proteins. Bioconjugation of antibodies usually uses lysine and cysteine residues via amide formation [34] and maleimide-thiol coupling [35, 36], respectively. However, primary amines and thiol groups are often found at unwanted sites of antibody molecule. Therefore, the bioconjugation of macromolecules to scFv may result in occlusion of antigen-binding sites, consequently diminishing their antigen-binding activity [37]. For monoclonal antibodies (mAbs), a cysteine residue containing a free thiol was sometimes introduced into a specific site to minimize the loss of antigen-binding activity [38–40]. This free thiol was effectively utilized for the conjugation of other molecules, such as anti-cancer drugs. Since mAbs have an Fc region that is not involved in antigen binding, the introduction of cysteine to the Fc region led to minimal changes in antigen binding capacity. In the case of scFv, there is no Fc region, making it non-trivial to identify an optimal site for cysteine introduction. Furthermore, such a free thiol on the small scFv may accelerate the aggregation of scFvs via disulfide bond formation, in addition to hydrophobic interactions.

To avoid this issue, we explored the site-specific HSA conjugation to scFv using click chemistry. Among the various click chemistries, our preference lies with the inverse electron-demand Diels-Alder reaction (IEDDA). This reaction is recognized for its remarkable site-selectivity, as it rapidly and efficiently couples tetrazine with trans-cyclooctene (TCO) without necessitating the use of a catalyst, setting it apart from other click chemistry reactions, such as copper catalyzed azide-alkyne cycloaddition (CuAAC) and strain-promoted azide-alkyne cycloadditon (SPAAC). Moreover, the IEDDA reaction demonstrates high stability and rapid reactivity, even under mild physiological pH conditions. These attributes have been applied numerous studies in biological conjugation [32, 41–45].

For site-specific bioconjugation of HSA and scFv, the site-specific introduction of a non-natural amino acid with a clickable functional group into scFv is necessary. In this context, careful selection of the bioconjugation site on the scFv for introducing a non-natural amino acid is crucial. It should avoid interfering with the complementary determining region (CDR) where the scFv binds to the antigen while also not impacting the FcRn-mediated recycling of HSA. This ensures that the scFv can effectively perform its function and conjugate without compromising HSA’s functionality [31, 46, 47].

To assess the structural integrity of the designed protein, methods have recently been known to predict protein structure using computational methods and find optimal site selection through scoring. So, we employed protein engineering software, including AlphaFold2 and PyRosetta. AlphaFold2 is a state-of-the-art software tool for predicting protein structures and has been increasingly utilized in protein structure prediction studies [48–52]. The use of AlphaFold2 allowed us to identify a suitable site for introducing a non-natural amino acid that does not affect the CDR region [52]. Subsequently, PyRosetta was employed for scoring to ensure that the introduced non-natural amino acid do not adversely impact the overall protein structure [48, 53]. This scoring mechanism aided in the selection of optimal candidate sites [48, 49, 53–57].

As a model protein, we used the 4D5scFv derived from Trastuzumab (Herceptin), which is employed in the treatment of human epidermal growth factor receptor 2 (HER2)-positive breast cancer. HER2 is a cell surface tyrosine kinase receptor overexpressed in various solid cancers. Notably, HER2 overexpression occurs in approximately 25–30% of breast cancer cases, and it is also observed in other solid cancers, including gastric cancer [58–60].

In this study, we identified candidate sites on the sequence of the model protein, 4D5scFv, suitable for site-specific conjugation without compromising its characteristics using AlphaFold2 and PyRosetta. Subsequently, to enable site-specific conjugation using IEDDA, we incorporated a phenylalanine analog with a hydrogen-substituted tetrazine group (frTet) using a pair of engineered *Methanococcus jannaschii* tyrosyl-tRNA/tyrosyl-tRNA synthetase (MjtRNT^Tyr^/MjTyrRS). Therefore, we hypothesized that through site-specific conjugation of HSA and 4D5scFv, we can extend the half-life without interfering antigen-binding affinity.

## Materials and methods

### Materials

*Escherichia coli* TOP10 competent cells (C404010) were purchased from Invitrogen (Karlsruhe, Germany). 4-(1,2,3,4-tetrazin-3-yl) phenylalanine (frTet) was purchased from WuXi Apptech (Shanghai, China). Bactotryptone (#211,705) and yeast extract (#212,750) were sourced from BD Biosciences (San Jose, CA, USA). Nickel-nitrilotriacetic acid (Ni-NTA) agarose resin (#30,210) was obtained from Qiagen (Germantown, MD, USA). Disposable PD-10 desalting columns (#GE17-0851-01) and Superdex 200 10/300 GL Increase columns were purchased from Cytiva (Marlborough, MA, USA). A steel target plate (#8,280,781) and Protein Standard II (#8,207,234) were obtained from Bruker (Billerica, MA, USA). Trifluoroacetic acid (TFA; 99%) was obtained from Daejung Chemicals & Metals (Gyeonggi, Korea). Immunoplates (96-well, flat, #32,396) were obtained from SPL Life Sciences (Gyeonggi, Korea). Recombinant human HER2 (#10,004-HCCH) was sourced from Sino Biological (Beijing, China). Tween-20 (#1,610,781) was obtained from Bio-Rad (Hercules, CA, USA). Skim milk (#SKI400) was purchased from BioShop (Burlington, ON, Canada). Horseradish peroxidase (HRP)-linked anti-rabbit IgG antibody (#7074) was procured from Cell Signaling 3Technology (Danvers, MA, USA). Hydrochloric acid (HCl; #001_00122) was obtained from Duksan Central Science (Seoul, Korea). L-(+)-arabinose (#A11921), disposable polypropylene columns (#29,924) and Zeba Spin Desalting Columns (7000 molecular weight cut-off (7 K MWCO), #89,882) were obtained from Thermo Fisher Scientific (Waltham, MA, USA). TCO-Cy3 was purchased from AAT Bioquest (Sunnyvale, CA, USA). TCO-PEG4-maleimide (TCO-PEG4-MAL) are purchased from FutureChem (Seoul, Korea). Vivaspin 6 centrifugal concentrators with a molecular weight cut-off (MWCO) of 10 and 50 kDa were purchased from Sartorius (Göttingen, Germany). All other chemical reagents were purchased from Sigma-Aldrich (St Louis, MO, USA) unless otherwise indicated.

### Computational evaluation of the frTet incorporation sites in 4D5scFv

To identify the optimal sites for frTet incorporation, a computational screening process was conducted. The model structure of 4D5scFv used in this analysis was generated using ColabFold, a web server that provides protein structure predictions of AlphaFold2 [52, 61]. The validation of the resulting model has been previously documented [46]. The selection of potential frTet incorporation sites within the structure was guided by several criteria: (1) Residues with a high solvent accessibility was chosen by calculating solvent-accessible surface area (SASA) using PyMOL (Version 2.4.1) (Schrödinger, New York, NY, USA); (2) Residues exhibiting stability when mutated into bulky aromatic ring residues were screened using PyRosetta (Pyrosetta4, the PyRosetta Team at Johns Hopkins University, Baltimore, MD, USA) [48]. Individual amino acid residues were mutated into either tyrosine (Y) or tryptophan (W), and then energy score was computed for each variant based on the energy score function; (3) Residues located within structured regions were excluded; (4) Residues located within or in proximity to the CDR were also excluded.

### Plasmid construction for expression of 4D5scFv variants

The plasmid of the wild-type 4D5scFv (pBAD_4D5scFv-WT) was obtained from the previous study [46]. In brief, the 4D5scFv protein consists of the V_H_ region at its N-terminal and the V_L_ region at its C-terminal, connected by a flexible linker of 15 amino acids (GGGGS)_3_ [62]. A hexahistidine-tag (His-tag) sequence was appended at the C-terminal for purification purposes. The DNA sequence encoding this protein was optimized using the ExpOptimizer software, developed by NovoPro Bioscience (Shanghai, China). The full amino acid and base pair sequence of 4D5scFv are listed in Supplementary Table S1.

After plasmid construction, we employed the site-directed mutagenesis polymerase chain reaction (PCR) technique to introduce amber codons at a specific site (position at 122, 125, 127, 177, or 178) identified through PyRosetta scoring using pBAD_4D5scFv-WT as a template to generate pBAD_4D5scFv_Amb plasmid variants (pBAD_4D5scFv-S122Amb, pBAD_4D5scFv-G125Amb, pBAD_4D5scFv-S127Amb, pBAD_4D5scFv-P177Amb, and pBAD_4D5scFv-G178Amb), respectively. The primer pairs used for PCR amplification are detailed in Supplementary Table S2.

### Expression and purification of 4D5scFv and 4D5scFv-frTet variants

For expression of 4D5scFv-frTet (4D5scFv-S122frTet, 4D5scFv-G125frTet, 4D5scFv-G125frTet, 4D5scFv-S127frTet, 4D5scFv-P177frTet, and 4D5scFv-G178frTet), pDule_C11RS plasmid and each of pBAD_4D5scFv_Amb plasmid variants (pBAD_4D5scFv-S122Amb, pBAD_4D5scFv-G125Amb, pBAD_4D5scFv-S127Amb, pBAD_4D5scFv-P177Amb, and pBAD_4D5scFv-G178Amb) were introduced into C321delA.exp *E. coli* host cells. The transformed *E. coli* cells were cultivated in a standard 2xYT medium supplemented with 100 µg/mL of ampicillin and 10 µg/mL of tetracycline and subjected to agitation at 200 rpm. When the optical density at 600 nm (OD_600_) reached 0.4, we introduced frTet into the culture at a final concentration of 1mM. Following induction, the culture temperature was lowered to 23 °C. The culture medium was subsequently incubated for 24 h. Then, cells were harvested through centrifugation at 8,000 rpm and 4 °C, and the resulting cell pellets were preserved at -80 °C. The expression of 4D5scFv-WT was performed as described previously [46]. In brief, 4D5scFv-WT was done using TOP10 *E. coli* host cells and procedure similar to that for 4D5scFv-frTet variants but without the additions of tetracycline and frTet. Pre-induction (BI) and post-induction (AI) samples were collected for each variant, followed by centrifugation at 13,000 rpm for 1 min and resuspension in PBS (pH 7.4) containing 2 M urea for subsequent SDS-PAGE analysis.

Purification of the His-tagged 4D5scFv-WT and 4D5scFv-frTet variants involves metal affinity chromatography using Ni-NTA. The cell pellets were solubilized in a lysis buffer (10 mM imidazole, 50 mM NaH_2_PO_4_, 300 mM NaCl, pH 8.0) supplemented with 1 mg/mL of lysozyme and 5 µg/mL of DNase. This mixture was incubated on ice for at least 5 min. After incubation, the solution underwent sonication with 1-second pulses and 2-second intervals, using an amplitude of 32% (500 W, 20 kHz) for a total duration of 15 min, followed by a repeat cycle after a 5-minute pause. The resulting mixture was then centrifuged at 10,000 rpm at 4 °C for 20 min. The supernatant was interacted with Ni-NTA agarose resin and maintained at 4 °C for 30 min. Post-incubation, the resin was loaded onto a polypropylene column with a filter, washed sequentially with a wash buffer (20 mM imidazole, 50 mM NaH2PO4, 300 mM NaCl, pH 8.0), and eluted with an elution buffer (250 mM imidazole, 50 mM NaH2PO4, 300 mM NaCl, pH 8.0). The eluted proteins were desalted with pH 7.4 PBS using a PD-10 desalting column. The purified proteins were stored at 4 °C until subsequent uses. Protein concentration was determined by the absorbance at 280 nm using a SynergyH1 microplate reader (BioTek, Winooski, VT, USA) according to Beer Lambert’s law (extinction coefficient is 50,100 M^− 1^cm^− 1^ for 4D5scFv-WT and 59,600 M^− 1^cm^− 1^ for 4D5scFv-frTet variants).

### Matrix-assisted laser desorption/ionization time-of-flight (MALDI-TOF) analysis

The purified 4D5scFv-WT and 4D5scFv-frTet variant (4D5scFv-G125frTet and 4D5scFv-G178frTet) and 4D5scFv-HSA conjugates (4D5scFv-G125-HSA and 4D5scFv-G178-HSA) were desalted using ZipTip C_18_ (Millipore, Billerica, MA, USA) and then mixed with a matrix solution containing sinapic acid (SA) saturated in TA30 (30% acetonitrile, 0.1% TFA in water) at a rate of 1:1 (v/v). The SA matrix saturated in ethanol solution was pre-deposited on the steel target plate. Subsequently, the prepared mixtures were subjected to analysis using a Microflex MALDI-TOF/MS device from Bruker Corporation (Billerica, MA, USA) in reflective, positive mode. Protein Standard II was used as a standard for calibration.

### Site-specific albumin conjugation to 4D5scFv-frTet variants via IEDDA reaction

HSA was purified using anionic exchange chromatography on a HiTrap Q HP column, with a flow rate of 0.2 mL/min, and monitored by absorbance at 280 nm using NGC Quest 10 Plus Chromatography System (Bio-Rad Laboratories Inc., Berkeley, CA, USA). The purified HSA was subsequently desalted using PBS (pH 7.0) and then subjected to a reaction with TCO-PEG4-MAL by thiol–maleimide coupling in PBS (pH 7.0) at a molar ratio of 1:4. After a 2-hour incubation period, the reaction mixture was desalted with PBS (pH 7.4) using a PD-10 column to eliminate unreacted TCO-PEG4-MAL linker and obtain the TCO-HSA conjugate. The purified 4D5scFv-frTet variants were then reacted with TCO-HSA at a molar ratio of 1:4 in PBS (pH 7.4) at 4 °C overnight. Following conjugation, the reaction mixture underwent anionic exchange column chromatography using the NGC Quest 10 Plus Chromatography System. Then metal affinity chromatography was performed using Ni-NTA. Molecular weight and purity assessments of eluted fractions were performed using sodium dodecyl sulfate–polyacrylamide gel electrophoresis (SDS-PAGE) analysis.

## 5,5’-Dithiobis(2-nitrobenzoic acid) (DTNB) assay

Purified HSA and TCO-HSA were buffer exchanged with PBS (pH 7.0). For the DTNB assay, 100 µL of Ellman’s reagent solution (12 mg of DTNB in 30 mL of 0.1 M sodium phosphate containing 1 mM EDTA, pH 8.0) was added to 100 µL of a 100 µM HSA sample, and the mixture was incubated for 5 min at room temperature [63]. Subsequently, the optical density of the reaction mixture was measured at 412 nm for the quantification of free thiol groups. Cysteine served as a standard for calibration, facilitating the conversion of optical density readings into thiol concentrations.

### Anti-HER2 enzyme-linked immunosorbent assay (ELISA) of 4D5scFv-WT and 4D5scFv-HSA conjugates

The efficacy of anti-HER2 targeting for the purified 4D5scFv-WT and 4D5scFv-HSA conjugates (4D5scFv-G125-HSA and 4D5scFv-G178-HSA) was assessed using an anti-HER2 enzyme-linked immunosorbent assay (ELISA). To initiate the assay, a recombinant HER2 antigen (50 ng, 100 µL/well) in a coating buffer (PBS, pH 7.4) was coated onto the immunoplate through overnight incubation at 4 °C. The plate underwent three washes with PBST (0.05% Tween 20 in PBS (pH 7.4)) at 200 µL/well. Subsequently, the plate was blocked by incubation with a blocking buffer (5% skim milk in PBST) at 200 µL/well at room temperature, followed by four washes. Afterwards, 4D5scFv-WT and 4D5scFv-HSA conjugates were prepared by diluting them 4-fold in a blocking buffer from 250 to 0.005 nM. Then, 100 µL was loaded into each well of the plate, and the plate was incubated at room temperature (*n* = 2). After incubation, unbound protein was removed by washing the plate four times. For detection, the plate was incubated with 100 µL/well of rabbit anti-His-tag antibody, diluted 1:1500 in blocking buffer. Following another round of incubation, the wells were washed four times. Finally, the plate was exposed to 100 µL/well of HRP-linked anti-rabbit IgG antibody, diluted 1:3000 in blocking buffer. After the subsequent washes, detection was carried out by adding 100 µL/well of TMB, and the reaction was halted using 2 M HCl. The quantification of bound proteins was achieved by measuring the absorbance at 450 nm.

### Pharmacokinetic study of 4D5scFv-WT and 4D5scFv-HSA conjugates in vivo

The pharmacokinetic study of 4D5scFv-WT and 4D5scFv-HSA conjugates (4D5scFv-G125-HSA and 4D5scFv-G178-HSA) in mice was conducted following the guidelines of the Animal Care and Use Committee of the Gwangju Institute of Science and Technology (GIST-2021-092). For this study, 2.5 µM of 4D5scFv-WT, or 4D5scFv-HSA conjugates in 200 µL of PBS at pH 7.4 were administered via the tail vein to 8-week-old female BALB/c mice (*n* = 5). Blood samples were collected via retro-orbital bleeding at specific time points: 5 min and 1 h for 4D5scFv-WT, and 5 min, 2, 4, 8, 12, 24, and 48 h for the 4D5scFv-HSA conjugates. After blood collection, serum was separated from the blood through centrifugation at 1,500 rpm at 4 °C for 10 min and stored at − 20 °C for subsequent use. Following serum separation, the serum concentration of 4D5scFv-WT and 4D5scFv-HSA conjugates was determined using anti-HER2 ELISA, as described above. Concentrations were calculated by interpolating the standard calibration curves.

## Results and discussions

### Preparation of 4D5scFv-WT and 4D5scFv-frTet variants

**Fig. 1:**
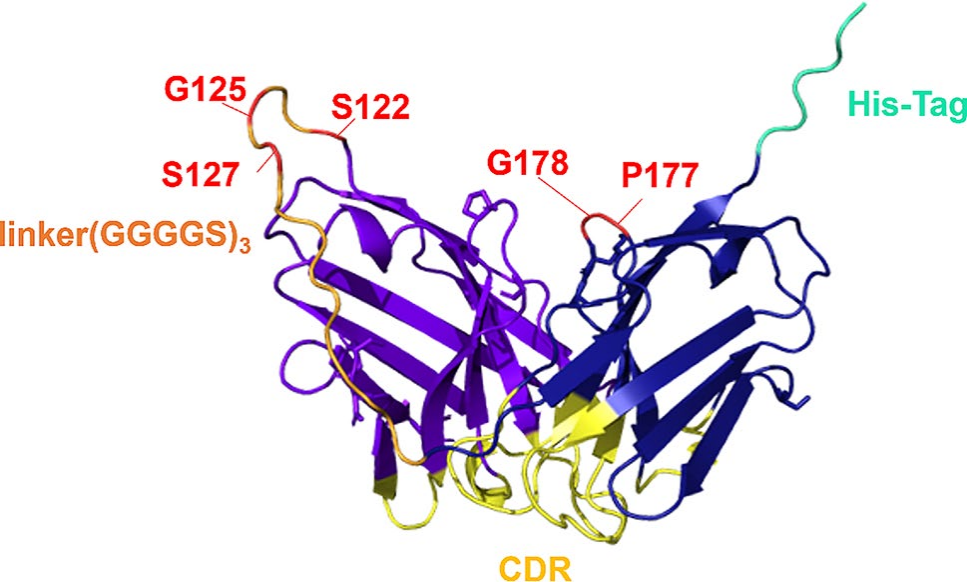
Annotated structure of 4D5scFv. Five selected candidate sites (red), CDR (yellow), His-tag (cyan), and linker (orange)

We genetically incorporated frTet into a specific site of 4D5scFv, to enable reaction with TCO-functionalized HSA. Candidate sites for frTet incorporation were selected through a comprehensive computational analysis on the model structure of 4D5scFv. Instead of relying on the reported crystal structure of the corresponding V_H_ and V_L_ regions (PDB ID: 1FVC) [64], we chose to work with the AlphaFold2-predicted scFv structure to better account for the presence of the fused linker region [52, 61]. As documented in prior research, the generated model exhibited a high degree of similarity with the 1FVC structure. Based on the 4D5scFv-WT structure (Fig. 1), we initially identified residues with high solvent accessibility. High solvent accessibility increases the likelihood of collisions, resulting in higher reactivity toward TCO. Subsequently, we focused on identifying residues least likely to disrupt the native structure upon frTet incorporation using PyRosetta [49, 57, 65]. To achieve this, we conducted point mutations into Y and W, which are natural amino acid analogues of frTet with a bulky aromatic group, for all candidate sites. We then compared the energy score of 4D5scFv-WT with those of the mutated variants and subsequently selected stabilizing variants. (Table S3). Lastly, we excluded residues located at or close to functionally or structurally important domains, including helices, sheets, CDRs, and disulfides. Five candidate sites (S122, G125, S127, P177, and G178) were selected. All candidates are situated away from the CDR. Particularly, G125 and S127 are located within the linker region.

### Preparation and characterization of 4D5scFv-WT and 4D5scFv-frTet variants

**Fig. 2:**
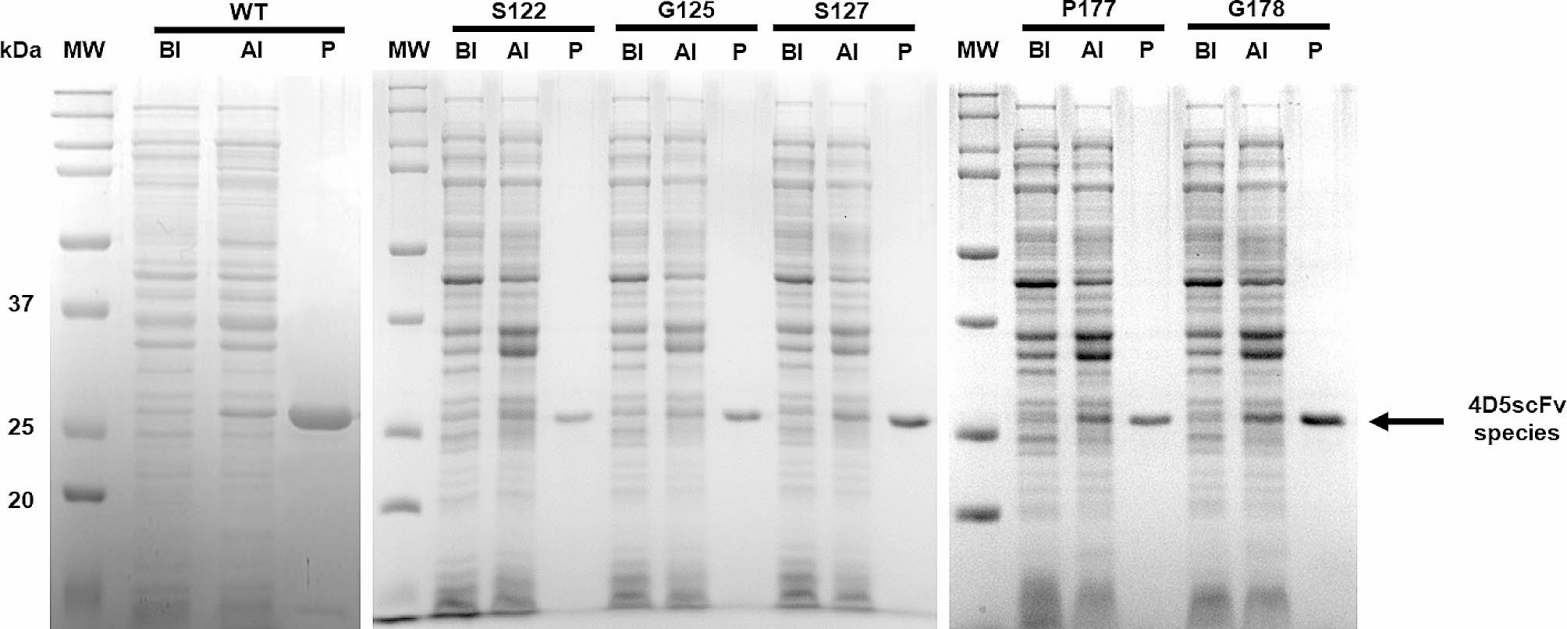
SDS PAGE analysis of expression and purification of 4D5scFv-WT; WT and 4D5scFv-frTet variants (S122; 4D5scFv-S122frTet, G125; 4D5scFv-G125frTet, S127; 4D5scFv-S127frTet, P177; 4D5scFv-P177frTet, and G178; 4D5scFv-G178frTet). Proteins were separated using a 5% acrylamide stacking gel, followed by a 12% linear gradient gel. Lanes MW: Bio-Rad SDS PAGE dual color molecular weight standards ladder. Lanes BI: pre-induction samples. Lanes AI: post-induction samples. Lanes P: purified protein samples

4D5scFv-WT and five 4D5scFv-frTet variants (4D5scFv-S122frTet, 4D5scFv-G125frTet, 4D5scFv-S127frTet, 4D5scFv-P177frTet, and 4D5scFv-G178frTet) were expressed in *E. coli* cells. In the SDS-PAGE analysis of cell lysates collected during expression (Fig. 2), bands became prominent for the post-induction samples (lanes AI), with molecular weights of 27 kDa, corresponding to those of 4D5scFv-WT and the 4D5scfv-frTet variants, respectively. Purification was performed using the interaction between Ni-NTA on agarose resin and the His-tag affinity tag on the C-terminus of each protein. In the SDS-PAGE analysis of the purified proteins (Fig. 2, lanes P), bands at the similar position as the 4D5scFv bands in the expression gel were detected. The production yields of purified 4D5scFv-WT, 4D5scFv-frTet variants (4D5scFv-S122frTet, 4D5scFv-G125frTet, 4D5scFv-S127frTet, 4D5scFv-P177frTet, and 4D5scFv-G178frTet) were 18.84 ± 3.81, 9.81 ± 2.93, 11.22 ± 1.92, 15.38 ± 5.43, 5.20 ± 2.74, and 12.58 ± 1.51 mg/L, respectively. Overall, these results demonstrate the successful expression and purification of the 4D5scFv-WT and 4D5scFv-frTet variants.

We analyzed the difference in molecular weight between the 4D5scFv-WT and the 4D5scFv-frTet variants (4D5scFv-G125frTet and 4D5scFv-G178frTet) using MALDI-TOF analysis (Fig. S1). The expected molecular weight of 4D5scFv-WT is 26,800 Da. Glycine (MW: 75 Da) at positions 125 and 178 was replaced with frTet (MW: 245 Da) in the selected 4D5scFv-frTet variants, leading us to anticipate a molecular weight of 26,970 Da, which is 170 Da higher than the 4D5scFv-WT. In the mass spectra of intact 4D5scFv-WT, 4D5scFv-G125frTet, and 4D5scFv-G178frTet, peaks were observed at 26,772, 26,942, and 26,943 Da, respectively. The calculated difference in molecular weight between 4D5scFv-WT and the 4D5scFv-frTet variants was 170 Da for 4D5scFv-G125frTet and 171 Da for 4D5scFv-G178frTet, respectively.

### Conjugation and purification of 4D5scFv-HSA

**Fig. 3:**
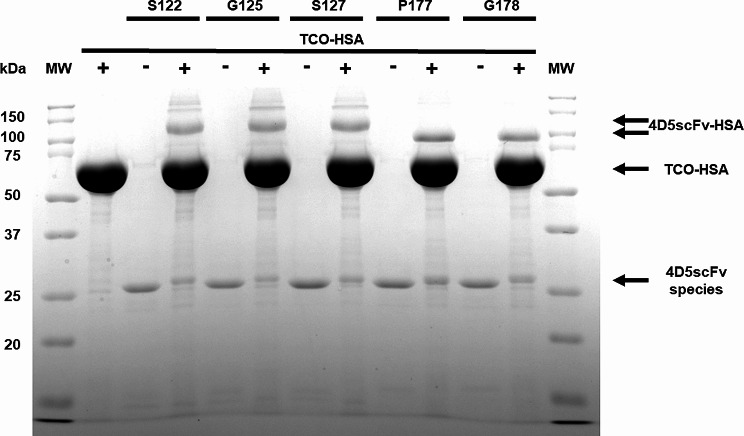
SDS PAGE analysis of 4D5scFv-HSA conjugation mixtures (S122; 4D5scFv-S122-HSA, G125; 4D5scFv-G125-HSA, S127; 4D5scFv-S127-HSA, P177; 4D5scFv-P177-HSA and G178; 4D5scFv-G178-HSA). Coomassie-stained protein gels of 4D5scFv variants incubated in the presence (+) or absence (-) of TCO-HSA

To prepare HSA-conjugated 4D5scFv, we utilized the heterobifunctional crosslinker TCO-PEG4-MAL. Initially, TCO-PEG4-MAL was employed to conjugate to the free cysteine at position 34 (Cys34) of HSA through a Michael addition reaction. Subsequently, TCO-HSA was conjugated to the purified 4D5scFv-frTet variants via the IEDDA reaction, resulting in the generation of 4D5scFv-HSA conjugates. The free thiol content of HSA was measured to be about 44% before the reaction and approximately 17% after linker conjugation. As a result, the TCO-HSA only accounts for about 27% of all HSAs (Fig. S2 A). Therefore, although scFv and HSA were reacted at a ratio of 1:4, the effective reaction ratio was approximately 1:1. We compared the HSA and TCO-HSA samples under reducing and non-reducing conditions, no discernible differences except band locations were observed (Fig. S2 B). The reaction mixtures underwent SDS-PAGE analysis (Fig. 3).

In the SDS-PAGE gel analysis, the bands corresponding to HSA-conjugated 4D5scFv-frTet variants (4D5scFv-S122frTet, 4D5scFv-G125frTet, 4D5scFv-S127frTet, 4D5scFv-P177frTet, and 4D5scFv-G178frTet) were observed within the molecular weight range of 100–150 kDa (Fig. 3). In trend of HSA conjugation yield for the 4D5scFv-frTet variants, the 4D5scFv-G125frTet and 4D5scFv-G178frTet variants exhibited the highest conjugation yield (Table S4) and were selected for further characterization.

**Fig. 4:**
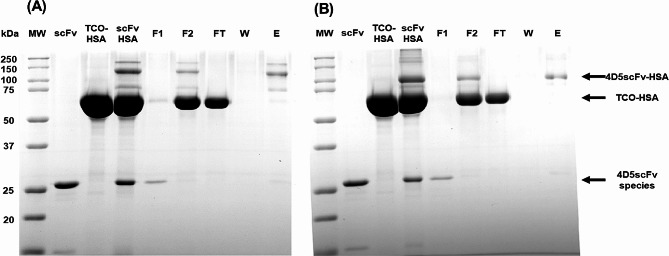
SDS PAGE analysis of conjugation and purification of 4D5scFv-HSA conjugation mixtures and anionic exchange chromatograms. 4D5scFv-G125-HSA is (**A**) and 4D5scFv-G178-HSA is (**B**). Lane F1: initial eluted fraction of anionic exchange chromatogram of 4D5scFv-HSA conjugation mixture. Lane F2: final eluted fraction of anionic exchange chromatogram of 4D5scFv-HSA conjugation mixtures (Fig. S3). Lanes FT: flow through fractions of metal affinity chromatograms of 4D5scFv-HSA conjugation mixtures after anionic exchange chromatography. Lanes W: washed fractions of metal affinity chromatograms of 4D5scFv-HSA conjugation mixtures after anionic exchange chromatography. Lanes E: eluted samples of metal affinity chromatograms of 4D5scFv-HSA conjugation mixtures after anionic exchange chromatography

However, there were detectable amounts of unconjugated 4D5scFv-frTet variants and residual TCO-HSA present after the conjugation process. To purify 4D5scFv-HSA conjugates, we employed a two-step purification strategy involving anion exchange chromatography and metal affinity chromatography using Ni-NTA. The eluted fractions from the chromatograms were subsequently analyzed by SDS-PAGE (Fig. 4). In lane E, it is evident that almost all unconjugated 4D5scFv-frTet variants and HSA were successfully removed during the chromatography step. In case of 4D5scFv-G125-HSA conjugate, there were some impurity bands (Fig. 4A). Although we purified HSA to remove HSA aggregates as much as possible before HSA conjugation to scFv, we found out the HSA multimer formation occurred during the conjugation and purification process of the scFv-HSA conjugate preparation. HSA tends to form dimers or oligomers via non-covalent bonds. HSA can also form covalent dimers, preserving the overall structure of the monomer [66]. If HSA were to form a dimer in the conjugation mixture condition and then conjugate with scFv, the resulting molecular weight would be approximately 160 kDa. The higher impurities observed in Fig. 4A are in the range of 250 − 150 kDa. Therefore, we speculate that they are impurities consisting of the dimeric form of scFv-HSA conjugate or the scFv-HSA conjugate with additional HSA. Although present in very small amounts, an impurity band can be observed at the 15 kDa position of the scFv band, a phenomenon also noted during the purification of the 4D5scFv variant. This impurity is also visible at the 15 kDa position in the scFv band lane in Fig. 4A. This suggests that some scFv species form dimers via non-covalent bond. The additional scFv seems separated from the scFv-HSA conjugate in the protein gel. If the free scFv impurity reacted with the TCO-HSA, its molecular weight would be approximately 80 kDa. Therefore, we speculate the protein band between 100 and 75 kDa seen in Fig. 4A corresponds to this impurity. The protein bands of three 4D5scFv-HSA conjugate variants (S122, G125, and S127) appeared at different locations from those of other two 4D5scFv-HSA conjugate variants (P177 and P178). Since the protein mobility can be influenced by other factors such as protein shape, we speculate that the HSA conjugation at different sites of 4D5scFv results in a distinct shape of the conjugate, leading to differences in protein mobility. Similar variations in protein mobility among the HSA-conjugated proteins were previously reported [65]. Furthermore, MALDI-TOF MS analysis was conducted for 4D5scFv-G125-HSA and 4D5scFv-G178-HSA, revealing the measured values of 94,080 and 94,351 Da, respectively, which are consistent with the expected mass of 94,010 Da (Fig. S1). These results suggest that a protein’s molecular weight and mobility in a gel may not precisely correspond.

### Anti-HER2 enzyme-linked immunosorbent assay (ELISA) of 4D5scFv-WT and 4D5scFv-HSA conjugates

**Fig. 5:**
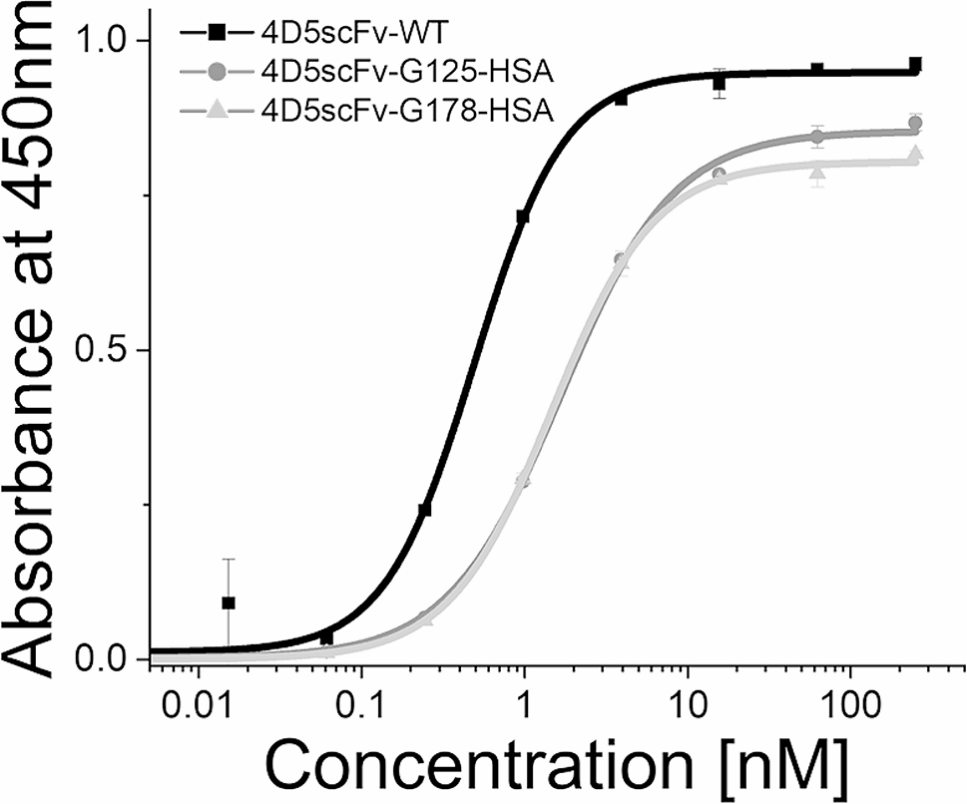
Anti-HER2 ELISA analysis of 4D5scFv-WT, 4D5scFv-G125-HSA, and 4D5scFv-G178-HSA. The purified proteins were loaded to plate initial concentration 250nM to 0.005nM, 4-fold dilution, respectively. Experiments were performed in triplicate, and error bars mean standard deviations

The binding affinities of 4D5scFv-WT and 4D5scFv-HSA conjugates (4D5scFv-G125-HSA and 4D5scFv-G178-HSA) against HER2 were investigated (Fig. 5). All the proteins exhibited a similar concentration-dependent curve. The half-maximal effective concentration (EC_50_) values for 4D5scFv-G125-HSA and 4D5scFv-G178-HSA were 1.67 and 1.47 nM, respectively, which showed only a slight deviation from that of 4D5scFv-WT (0.49 nM).

HSA binding to 4D5scFv resulted in a mild decrease in anti-HER2 binding affinity, probably due to the steric hindrance caused by the bulky HSA during the binding of 4D5scFv-HSA conjugates to HER2. There was also some decrease in the maximum values of 4D5scFv-HSA conjugates in anti-HER2 binding compared to that of 4D5scFv-WT (Fig. 5), probably because the conjugated HSA causes the reduced accessibility of the epitope (the His-tag in the 4D5scFv) by the anti His-tag antibody in the ELISA assay. These results suggest that HSA conjugation did not cause significant distortions in the 4D5scFv structure, as expected from previous computational predictions.

### Pharmacokinetics properties

**Fig. 6:**
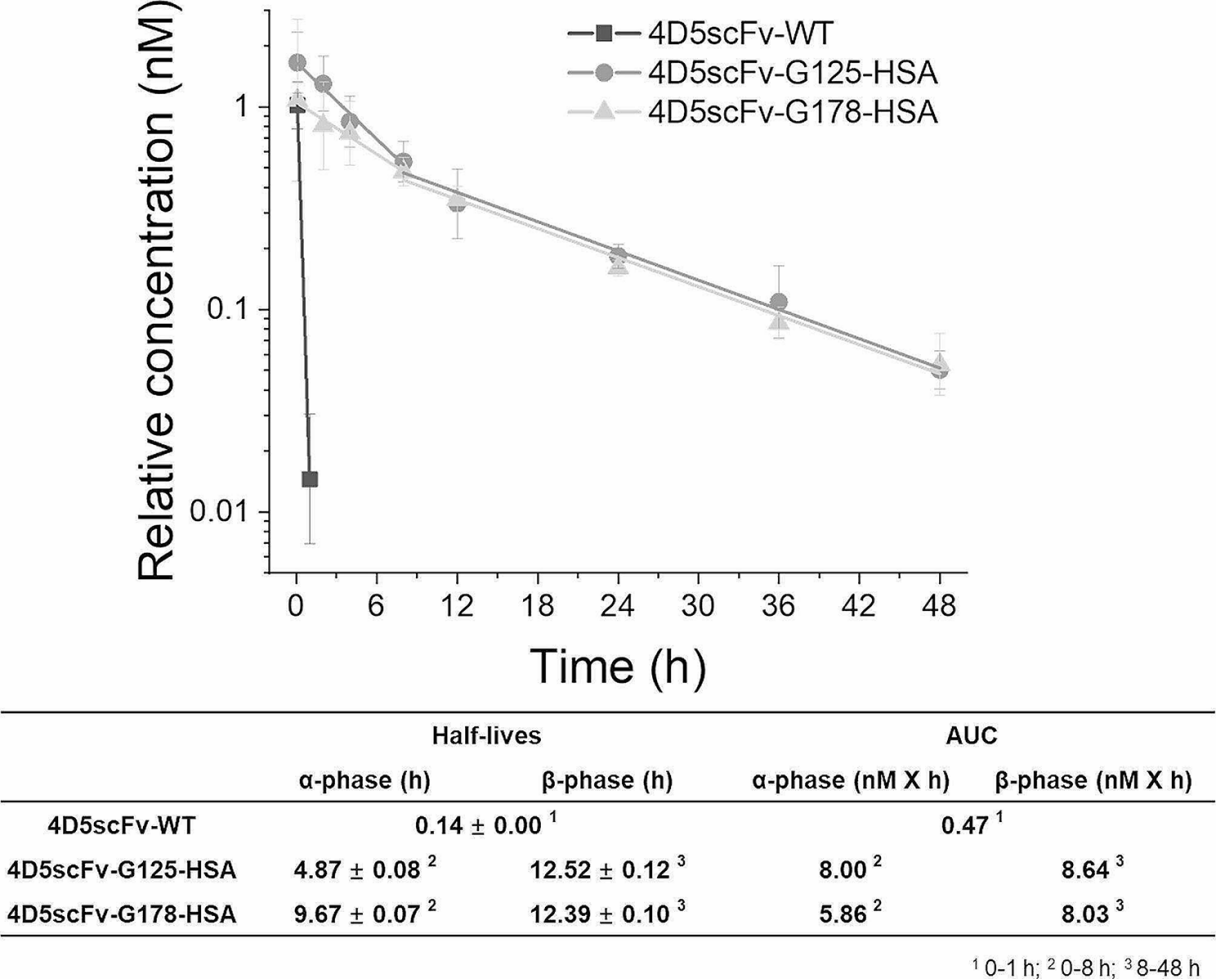
Pharmacokinetic profiles of 4D5scFv-WT, 4D5scFv-G125-HSA, and 4D5scFv-G178-HSA. Samples were injected into BALB/c mice (*n* = 5). Anti-HER2 ELISA analysis of the residual 4D5scFv-WT and 4D5scFv-HSA conjugates were measured at different time points (4D5scFv-WT; 5 min, 1 h and 4D5scFv-HSA conjugates; 5 min, 2, 4, 8, 12, 24, 36, and 48 h). α-phase and β-phase indicate the serum half-lives in the early phase (0 to 8 h) and late phase (8 to 48 h), respectively

To evaluate the pharmacokinetic effects of HSA conjugated 4D5scFv, we intravenously administered a single dose of either 4D5scFv-WT or 4D5scFv-HSA conjugates (4D5scFv-G125-HSA and 4D5scFv-G178-HSA) to BALB/c mice (*n* = 5). After collecting blood samples from each mouse at different time points, we separated the serum and measured the concentration of each 4D5scFv-WT or 4D5scFv-HSA conjugates in the serum using above anti-HER2 ELISA analysis. As previously reported [30–32], 4D5scFv-WT exhibited rapid clearance, with a serum half-life of 0.14 h (Fig. 6). In the case of 4D5scFv-HSA conjugates, the half-lives were observed to the early phase (0 to 8 h) and the late phase (8 to 48 h). In the early phase, the serum half-lives of 4D5scFv-G125-HSA and 4D5scFv-G178-HSA were measured at 4.87 and 9.67 h, respectively. In the late phase, the serum half-lives were determined to be 12.52 and 12.39 h, respectively. The calculated Cmax for 4D5scFv-WT was 1.02 ± 0.28 nM, while those of 4D5scFv-G125-HSA and 4D5scFv-G178-HSA were 1.65 ± 0.66 and 1.08 ± 0.52 nM, respectively. The area under curve (AUC) of 4D5scFv-WT was calculated as 0.47. For 4D5scFv-G125-HSA, the alpha phase was calculated as 8.00, and the beta phase was calculated as 8.64. Regarding 4D5scFv-G178-HSA, the alpha phase was calculated as 5.86, and the beta phase was calculated as 8.03. These results demonstrate that the conjugation of HSA significantly increased the half-life of 4D5scFv, by more than 85-fold. We believe that the increased half-life of 4D5scFv upon HSA conjugation is mainly attributed to reduced renal filtration. Small proteins (smaller than 50 kDa) such as scFv (about 27 kDa) are effectively eliminated through renal filtration [67]. Therefore, the conjugation of HSA (about 67 kDa) to 4D5scFv is expected to reduce renal filtration, leading to an increase in serum half-life. We also investigated whether FcRn-mediated recycling contributed to the increase in serum half-life via FcRn binding assays (Fig. S4). In case of human FcRn (hFcRn), both 4D5scFv-G125-HSA and 4D5scFv-G178-HSA exhibited the FcRn binding comparable to unmodified HSA (Fig. S5). However, we could not obtain any significant binding of 4D5scFv-G125-HSA and 4D5scFv-G178-HSA to murine FcRn (mFcRn) (data not shown). Therefore, the contribution of FcRn-mediated recycling to the serum half-life extension does not seem significant in mice. However, it has been reported that protein conjugation to the free thiol (cysteine34) of HSA does not compromise the binding affinity of HSA to hFcRn [65, 68]. There was a positive correlation between the in vitro relative FcRn binding affinity and in vivo serum half-life among the HSA-conjugated proteins [68]. Since HSA is known to have substantially lower binding affinity for mFcRn compared to the binding between hFcRn and HSA, we expect that the serum half-life of 4D5scFv-HSA conjugates will be longer in humans, as in the case of the albumin-fused glucagon-like peptide 1 (Albiglutide) used for type II diabetes treatment.

## Conclusions

Although antibody fragments, including scFv, hold great promise for treating diseases such as cancer and immune system disorders, scFv’s short blood circulation time has limited its clinical application. Therefore, we prepared HSA-conjugated scFv using an effective site-specific conjugation strategy to extend the serum half-life of scFv. HSA’s biocompatibility and long serum half-life made it an attractive choice as a safe and effective serum half-life extension agent for therapeutic proteins.

We devised an effective strategy for conjugating biomacromolecules to scFv, involving a computational screening process to predict optimal conjugation sites, followed by site-specific conjugation via the IEDDA reaction. The resulting 4D5scFv-HSA conjugates retained binding affinity comparable to 4D5scFv-WT, with a slight increase (3-fold) in the EC_50_ value observed in ELISA. Considering the high conjugation yield of over 70% and the highly preserved binding affinity after conjugation, this conjugation strategy can be non-destructive and efficient. Moreover, as expected, the 4D5scFv-HSA variants showed an approximately 85-fold longer serum half-life than 4D5scFv-WT in the pharmacokinetic profile. However, it is noteworthy that the serum half-life of the original anti-HER2 mAb (trastuzumab) in mice is longer than 10 days [69], substantially greater than those of the 4D5scFv-HSA variants. It is still challenging for HSA conjugation to recapitulate the reduced serum half-life of scFvs compared to original mAbs. Compared to mAbs, scFvs are relatively unstable and tend to aggregate. Therefore, although the conjugation of HSA prolongs the serum half-life of scFvs to some extent, many issues such as poor stability should be resolved for wider clinical applications of scFv-based drugs. This strategy/workflow described in this study shows potential as a versatile platform for utilizing other valuable biological macromolecules and expanding their potential clinical applications.

### Electronic supplementary material

Below is the link to the electronic supplementary material.

Supplementary Material 1

## Data Availability

The datasets used and/or analyzed during the current study are available from the corresponding author on reasonable request.

## Abbreviations

2xYT: 2x Yeast Extract Tryptone medium
4D5scFv: Single-chain variable fragment derived from the monoclonal antibody, trastuzumab
4D5scFv-HSA: Conjugated 4D5scFv and human serum albumin
Amb: Amber codon
CDR: Complementary determining region
CuAAC: Copper catalyzed azide-alkyne cycloaddition
DTNB: 5,5’-Dithiobis(2-nitrobenzoic acid)
*E. coli*: *Escherichia coli*
EC_50_: Half maximal effective concentration
ELISA: Enzyme-linked immunosorbent assay
frTet: 4-(1,2,3,4-tetrazin-3-yl) phenylalanine
FcRn: Neonatal Fc receptor
HER2: Human epithermal growth factor 2
HSA: Human serum albumin
HRP: Horseradish peroxidase
IEDDA: Inverse electron-demand Diels-Alder reaction
mAb: Monoclonal antibody
MALDI-TOF: Matrix-assisted laser desorption/ionization time-of-flight
mFcRn: Mouse neonatal Fc receptor
Ni-NTA: Nickel-nitrilotriacetic acid
OD: Optical density
PBS: Phosphate buffered saline
PCR: Polymerase chain reaction
PEG: Polyethyleneglycol
SASA: Solvent-accessible surface area
scFv: Single-chain variable fragment
SDS-PAGE: Sodium dodecyl sulfate–polyacrylamide gel electrophoresis
SPAAC: Strain-promoted azide-alkyne cycloaddition
TCO: Trans-cyclooctene
TCO-Cy3: Cy3 trans-cyclooctene
TCO-PEG4-MAL: TCO-PEG4-maleimide heterobifunctional crosslinker
TMB: 3,3′,5,5′-Tetramethylbenzidine
TFA: Trifluoroacetic acid
WT: Wild type
