# Supplementary material for: Enhanced therapeutic potential of antibody fragment via IEDDA-mediated site-specific albumin conjugation
Source: J Biol Eng. 2024 Apr 4;18:23. doi: 10.1186/s13036-024-00418-3 (PMC10996255; doi:10.1186/s13036-024-00418-3)
Supplement: Supplementary file 1 — Supplementary Material 1 [file 13036_2024_418_MOESM1_ESM.docx]

**Supplementary Information**

**Enhanced Therapeutic Potential of Antibody Fragment via IEDDA-mediated Site-Specific Albumin Conjugation**

Eun Byeol Go^1,#^, Jae Hun Lee^1,#^, Jeong Haeng Cho^2,3,#^, Na Hyun Kwon^1,#^, Jong-il Choi^3^, Inchan Kwon^1,*^

^1^School of Materials Science and Engineering, Gwangju Institute of Science and Technology (GIST), Gwangju 61005, Republic of Korea

^2^ProAbTech, Gwangju 61005, Republic of Korea

^3^Department of Biotechnology and Bioengineering, Interdisciplinary Program for Bioenergy and Biomaterials, Chonnam National University

^#^ These authors equally contribute to this manuscript.

^*^ Corresponding author.

School of Materials Science and Engineering, Gwangju Institute of Science and Technology, Gwangju 61005, Republic of Korea

Tel.: +82 62-715-2312

Fax: +82 62-715-2304

E-mail: inchan@gist.ac.kr (I. Kwon).

**Table S1** Full amino acid and base pair sequence of 4D5scFv-WT. V_H_ region are colored red, V_L_ region are colored blue.

| Amino acid sequence |
| --- |
| MGEVQLVESGGGLVQPGGSLRLSCAASGFNIKDTYIHWVRQAPGKGLEWVARIYPTNGYTRYADSVKGRFTISADTSKNTAYLQMNSLRAEDTAVYYCSRWGGDGFYAMDYWGQGTLVTVSS  **GGGGSGGGGSGGGGS**  DIQMTQSPSSLSASVGDRVTITCRASQDVNTAVAWYQQKPGKAPKLLIYSASFLYSGVPSRFSGSRSGTDFTLTISSLQPEDFATYYCQQHYTTPPTFGQGTKVEIK**HHHHHH** |
| Base pair sequence |
| CCATG(Start)GGTGAAGTTCAGCTGGTTGAATCTGGCGGTGGTCTGGTTCAGCCGGGTGGTTCTCTGCGTCTGAGCTGTGCTGCTTCCGGTTTTAACATTAAAGATACTTACATTCACTGGGTACGTCAGGCCCCGGGTAAAGGTCTGGAATGGGTTGCTCGCATCTACCCGACTAACGGCTACACCCGTTACGCGGACTCTGTCAAAGGTCGTTTTACTATTAGCGCTGATACCAGCAAAAACACGGCGTACCTGCAAATGAACTCCCTGCGTGCAGAAGATACCGCTGTCTACTACTGTAGCCGTTGGGGCGGTGACGGTTTTTACGCTATGGACTATTGGGGTCAGGGCACCCTGGTAACCGTATCTTCT  **GGTGGTGGTGGTTCTGGTGGTGGTGGTTCTGGTGGTGGTGGCTCC**  GACATCCAGATGACCCAGAGCCCGTCCTCTCTGTCTGCATCTGTTGGTGACCGTGTCACCATTACTTGCCGCGCCTCTCAGGATGTAAATACCGCTGTTGCGTGGTATCAACAGAAACCGGGCAAAGCTCCAAAGCTGCTGATTTACTCTGCTTCTTTCCTGTACTCTGGTGTTCCGTCTCGTTTCTCCGGTTCCCGTAGCGGCACTGACTTTACCCTGACCATCAGCAGCCTGCAACCGGAGGACTTCGCAACCTACTACTGCCAGCAGCACTACACTACCCCGCCGACCTTCGGTCAGGGTACTAAAGTGGAAATTAAGCACCATCACCACCACCATTAA(Stop) |

**Table S2** Primer pairs sequence. The top sequence in each primer sequence is the forward primer, the second sequence is the reverse primer.

| **Variants**  **name** | **Residue number** | **Amino acid** | **primer sequence** |
| --- | --- | --- | --- |
| **Q15** | **15** | **GLN** | 5'-ctggcggtggtctggtttagccgggtg-3’  5'-cacccggctaaaccagaccaccgccag-3' |
| **S87** | **87** | **SER** | 5'-ggcgtacctgcaaatgaactagctgcgtgcagaagat-3’  5'-atcttctgcacgcagctagttcatttgcaggtacgcc-3' |
| **R89** | **89** | **ARG** | 5'-tacctgcaaatgaactccctgtaggcagaagataccgctgtctac-3’  5'-gtagacagcggtatcttctgcctacagggagttcatttgcaggta-3' |
| **Q114** | **114** | **GLN** | 5'-cgctatggactattggggttagggcaccct-3’  5'-agggtgccctaaccccaatagtccatagcg-3' |
| **S122** | **122** | **SER** | 5'-ggcaccctggtaaccgtatcttagggtggtggtggt-3’  5'-accaccaccaccctaagatacggttaccagggtgcc-3' |
| **G125** | **125** | **GLY** | 5'-gtaaccgtatcttctggtggttagggttctggtggtggtggt-3’  5'-accaccaccaccagaaccctaaccaccagaagatacggttac-3' |
| **S127** | **127** | **SER** | 5'-cgtatcttctggtggtggtggttagggtggtggtggtt-3’  5'-aaccaccaccaccctaaccaccaccaccagaagatacg-3' |
| **S146** | **146** | **SER** | 5'-gatgacccagagcccgtagtctctgtctgcatct-3’  5'-agatgcagacagagactacgggctctgggtcatc-3' |
| **P177** | **177** | **PRO** | 5'-cgctgttgcgtggtatcaacagaaatagggcaaagctcca-3’  5'-tggagctttgccctatttctgttgataccacgcaacagcg-3' |
| **G178** | **178** | **GLY** | 5'-gcgtggtatcaacagaaaccgtagaaagctccaaagctgctgatt-3’  5'-aatcagcagctttggagctttctacggtttctgttgataccacgc-3' |
| **K179** | **179** | **LYS** | 5'-gtatcaacagaaaccgggctaggctccaaagctgctgattt-3’  5'-aaatcagcagctttggagcctagcccggtttctgttgatac-3' |
| **K244** | **244** | **LYS** | 5'-gtcagggtactaaagtggaaatttagcaccatcacc-3’  5'-ggtgatggtgctaaatttccactttagtaccctgac-3' |

**Table S3** Amino acid mutation coring of 4D5scfv variants using PyRosetta. After PyRosetta scoring, we selected 12 sites with a mutation energy score difference of 10 (wild-type score: 321.119), less from the wild type, excluding the CDR region, secondary structure, N-terminus, C-terminus, and the His-tag site. Among these, we further narrowed down our selection to the top 5 sites with a solvent accessibility of 80% or more (red).

| **Variants**  **name** | **Residue number** | **Amino acid** | ***Y mutation score*** | | ***W mutation score*** | | **Solvent accessibility (%)** | **Region** |
| --- | --- | --- | --- | --- | --- | --- | --- | --- |
|  |  |  | **Score** | **Deviation** | **Score** | **Deviation** |  |  |
| **Q15** | ***15*** | ***GLN*** | 320.4787 | 0.64027 | 322.8493 | 1.730285 | 70 | V_H_ |
| **S87** | ***87*** | ***SER*** | 321.8043 | 0.685291 | 323.9435 | 2.824543 | 50 | V_H_ |
| **R89** | ***89*** | ***ARG*** | 321.9117 | 0.792723 | 323.1212 | 2.002219 | 53 | V_H_ |
| **Q114** | ***114*** | ***GLN*** | 316.0304 | 5.088595 | 317.0127 | 4.106267 | 50 | V_H_ |
| **S122** | ***122*** | ***SER*** | 322.2008 | 1.081831 | 322.612 | 1.492976 | 93 | V_H_ |
| **G125** | ***125*** | ***GLY*** | 327.5827 | 6.463661 | 329.7311 | 8.612122 | 93 | linker |
| **S127** | ***127*** | ***SER*** | 322.955 | 1.836021 | 324.6238 | 3.504805 | 96 | linker |
| **S146** | ***146*** | ***SER*** | 322.5788 | 1.459825 | 323.4973 | 2.37832 | 63 | V_L_ |
| **P177** | ***177*** | ***PRO*** | 324.1598 | 3.040772 | 326.3442 | 5.225208 | 82 | V_L_ |
| **G178** | ***178*** | ***GLY*** | 327.0097 | 5.890653 | 329.8572 | 8.738239 | 95 | V_L_ |
| **K179** | ***179*** | ***LYS*** | 322.2758 | 1.156783 | 324.1933 | 3.07426 | 66 | V_L_ |
| **K244** | ***244*** | ***LYS*** | 321.5192 | 0.400216 | 324.3402 | 3.221235 | 54 | V_L_ |

**Table S4** 4D5scFv variants production yield and conjugation yield. Production yield was calculated in the triplet.

| **4D5scFv variants** | **Production Yield (mg/L)** | **Conjugation Yield with TCO-HSA (%)** |
| --- | --- | --- |
| 4D5scFv-WT | 18.84 ± 3.81 |  |
| 4D5scFv-S122frTet | 9.81 ± 2.93 | 62.12 |
| 4D5scFv-G125frTet | 11.22 ± 1.92 | 76.68 |
| 4D5scFv-S127frTet | 15.38 ± 5.43 | 73.05 |
| 4D5scFv-P177frTet | 5.20 ± 2.74 | 62.60 |
| 4D5scFv-G178frTet | 12.58 ± 1.51 | 74.85 |

**
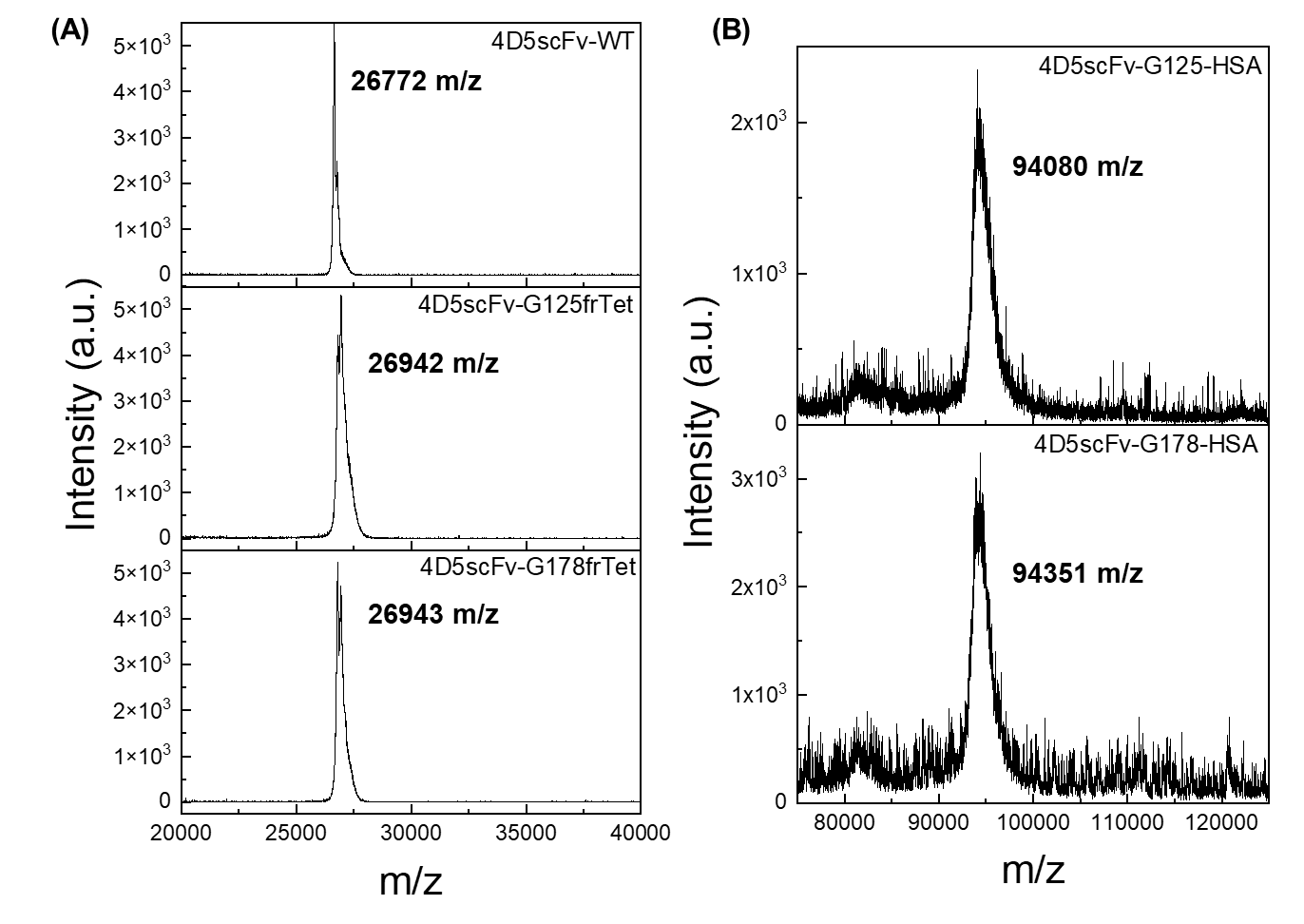
**

**Fig. S1** MALDI-TOF analysis of purified 4D5scFv-WT, 4D5scFv-G125frTet and 4D5scFv-G178frTet (A) and purified 4D5scFv-G125-HSA and 4D5scFv-G178-HSA (B).


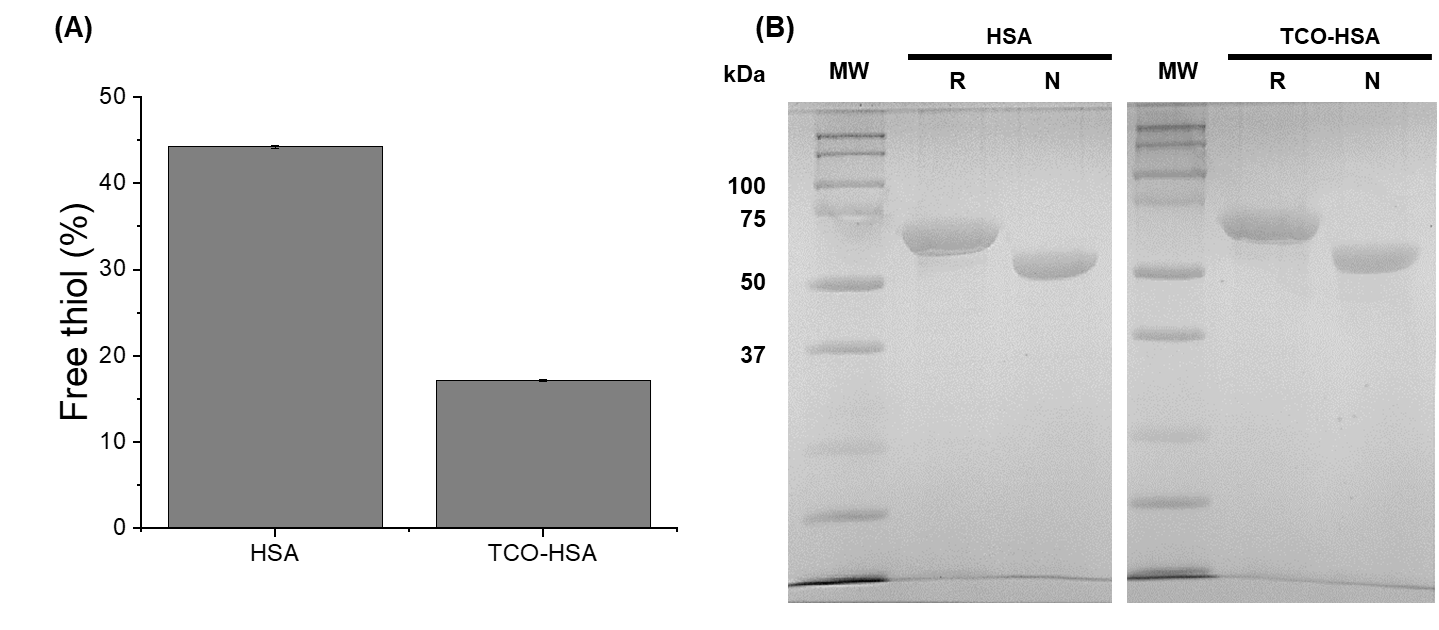


**Fig. S2** (A) Free thiol content of HSA and TCO-HSA via DTNB assay (n=3). Cysteine served as a standard for calibration. (B) SDS PAGE analysis of HSA and TCO-HSA under reducing and non-reducing conditions. Lanes MW: Bio-Rad SDS PAGE dual color molecular weight standards ladder. Lanes R: under reducing condition. Lanes N: under non-reducing condition.


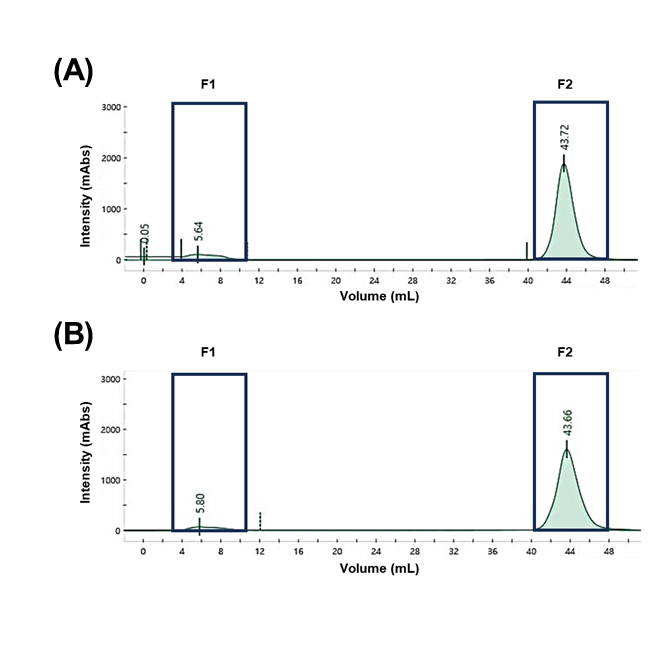


**Fig. S3** Anionic exchange chromatography of 4D5scFv-G125-HSA (A) and 4D5scFv-G178-HSA (B). All samples injected at least 5mL volume after conjugation. F1 is initial eluted fraction of anionic exchange chromatogram of 4D5scFv-HSA conjugation mixture. F2 is final eluted fraction of anionic exchange chromatogram of 4D5scFv-HSA conjugation mixture.

**FcRn binding assay of HSA and 4D5scFv-HSA variants**

**Materials and Methods**

Binding affinities of HSA, 4D5scFv-G125-HSA, and 4D5scFv-G178-HSA against human FcRn were investigated. Human FcRn tagged with Strep-Tag II (#FCM-H528, ACROBiosystems, DE, USA) was immobilized on Strep-Tactin®XT 4Flow® resin (#2-5010-025, IBA Lifesciences GmbH, Göttingen, Germany) at a ratio of 1 nmol of hFcRn per 1 mL of resin. After incubation at 4°C, unbound hFcRn was washed with PBS (pH 6.0) using a centrifuge column. The hFcRn-immobilized Strep-Tactin resin was then mixed with an equal concentration of HSA and 4D5scFv-HSA variants (>4 μM) in PBS (pH 6.0) and shaken at 4°C using a rotation shaker. After washing with PBS (pH 6.0) to remove nonspecific binding of HSA, proteins were eluted using PBS (pH 7.4). Eluted proteins were quantified by measuring absorbance. Data are represented as mean ± standard deviation (SD) values. One-way analysis of variance (ANOVA) with Bonferroni's test was performed to calculate the statistical significance of differences. Data visualization and statistical analysis were conducted using OriginPro (Version 2023, OriginLab Corporation, Northampton, MA, USA).

**Results and Discussion**

The FcRn binding behavior of HSA and 4D5scFv-HSA variants was investigated using hFcRn-immobilized resin, as illustrated in Figure S4. HSA and the variants were incubated with the hFcRn-bound resin at pH 6.0 and subsequently eluted for quantification at pH 7.4. This pH shift is crucial since the interaction between albumin and FcRn weakens significantly at neutral pH compared to weakly acidic conditions. As depicted in Figure S5, HSA and the two 4D5scFv-HSA variants were detected in the elution in the presence of hFcRn but were undetectable in its absence, indicating the specific binding between HSA and hFcRn. The two 4D5scFv-HSA variants showed comparable binding affinity to HSA, with no statistically significant differences from each other. This suggests that there may be similar effects to HSA from FcRn binding in humans. However, considering the inherent limitations of the experimental setup compared to more precise techniques such as ELISA, further investigation is required to see whether HSA and the 4D5scFv-HSA variants possess identical binding affinities.


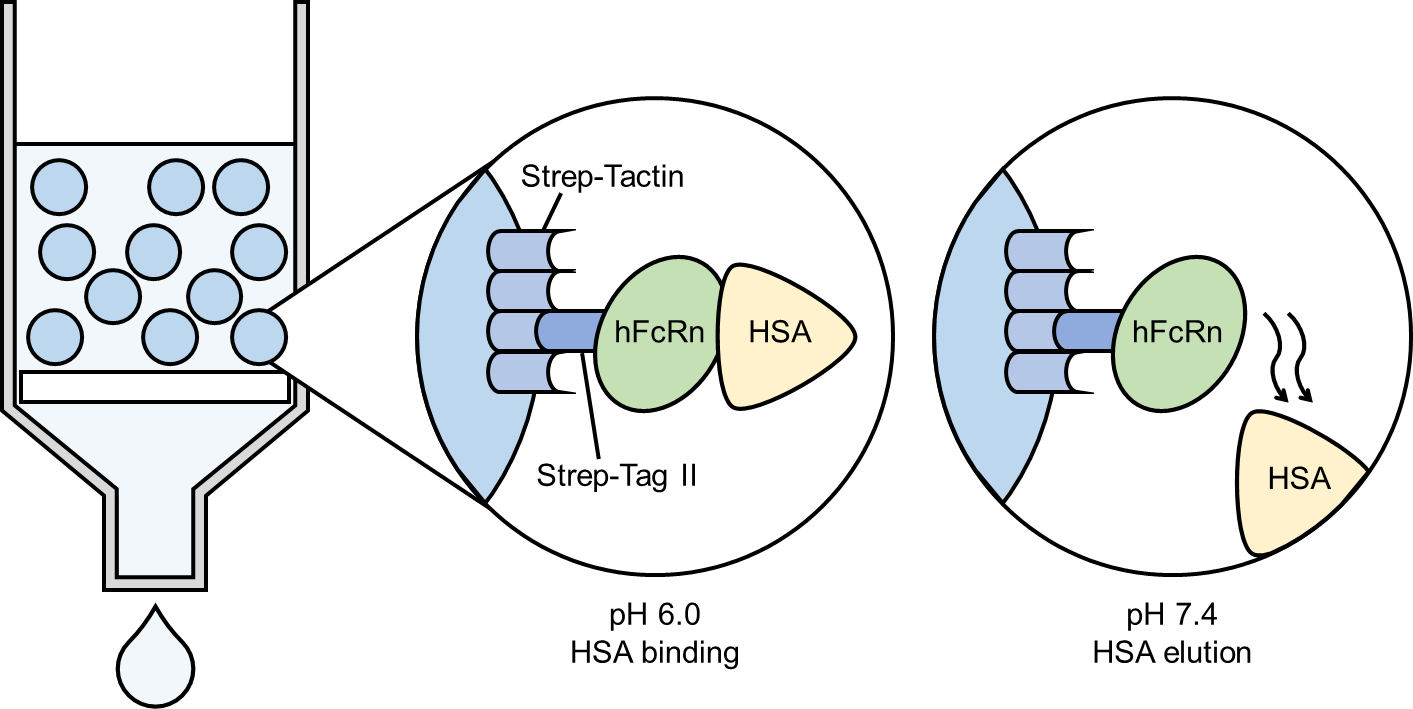


**Fig. S4** FcRn binding experiment scheme.

**
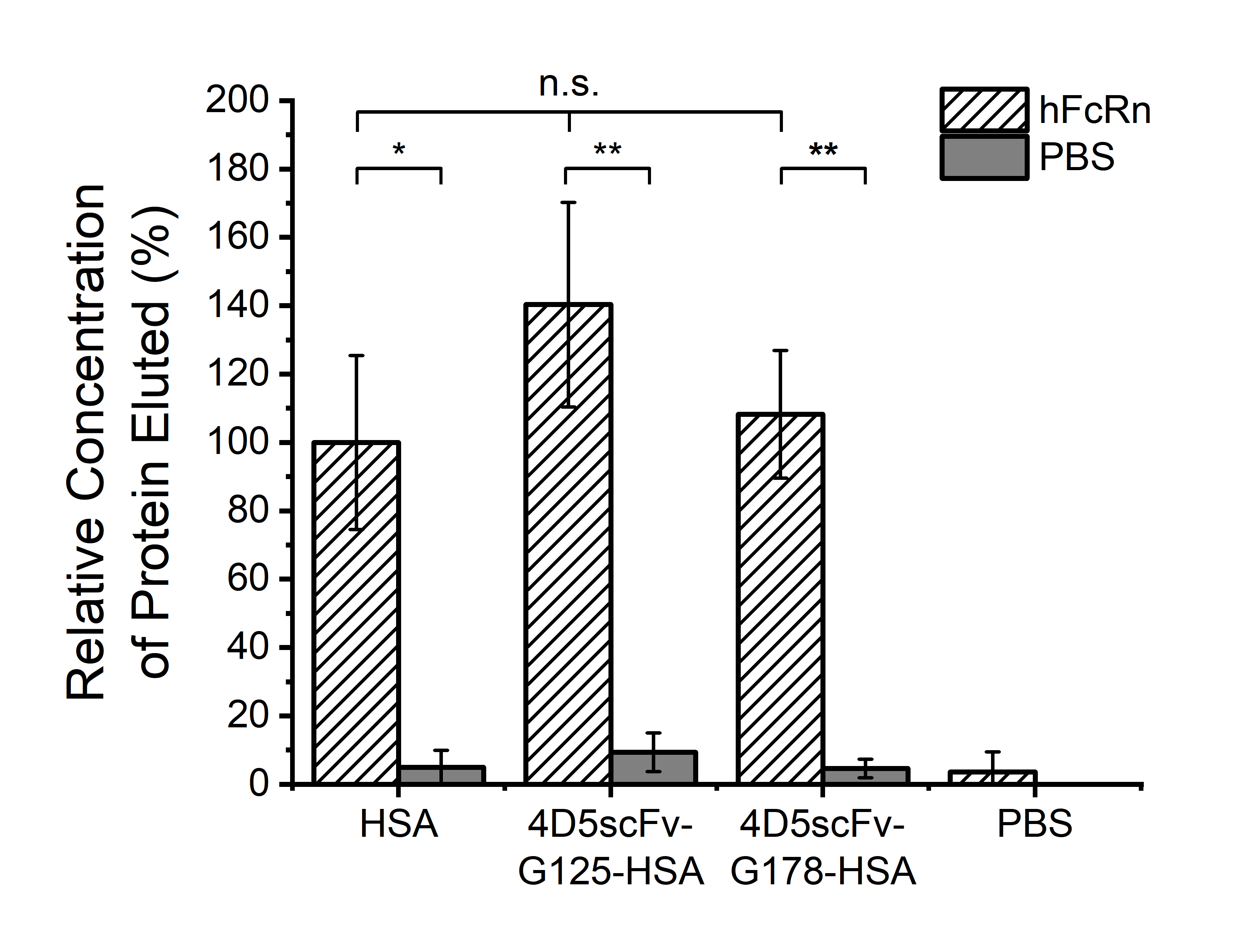
**

**Fig. S5** The hFcRn binding assays of HSA, 4D5scFv-G125-HSA, and 4D5scFv-G178-HSA are depicted. The data are presented as relative concentrations with the average value of HSA set to 100%. Each experimental group consists of n=4 replicates, while negative controls have n=2 replicates. PBS is another negative control where the experiment was performed in absence of any HSA. Results are shown as mean ± SD. Statistically significant differences were determined using one-way ANOVA, with p < 0.01 denoted as **, p < 0.05 as *, and p > 0.05 as n.s. (not significant).
